# Supplementary material for: Stakeholder theory and management: Understanding longitudinal collaboration networks
Source: PLoS One. 2021 Oct 14;16(10):e0255658. doi: 10.1371/journal.pone.0255658 (PMC8516199; doi:10.1371/journal.pone.0255658)
Supplement: Attachment — Submitted filename: Response to Reviewers.docx [file pone.0255658.s002.docx]

**Response to Reviewers**

The authors highly appreciate the reviewers’ insightful and helpful comments to improve the manuscript, and they would like to thank them for this great opportunity. Please see below our detailed response to the changes required. All line numbers in this document refer to the manuscript file while track changes are showing.

**Editors Comments:**

***1. Comment: Please ensure that your manuscript meets PLOS ONE's style requirements, including those for file naming. The PLOS ONE style templates can be found at***

***https://journals.plos.org/plosone/s/file?id=wjVg/PLOSOne_formatting_sample_main_body.pdf and***

***https://journals.plos.org/plosone/s/file?id=ba62/PLOSOne_formatting_sample_title_authors_affiliations.pdf***

The manuscript has been revised according to the guidelines listed in the above two links. For instance, see the updated affiliations at the beginning of the manuscript (lines 6-14). Also, the heading font size has been changed to 16 for heading 1, 14 to heading 2, etc.

***2. Comments: We note that you have indicated that data from this study are available upon request. PLOS only allows data to be available upon request if there are legal or ethical restrictions on sharing data publicly.***

 The dataset has now been submitted during the second revision and will be available.

***3. Comment: In your revised cover letter, please address the following prompts:***

***a) If there are ethical or legal restrictions on sharing a de-identified data set, please explain them in detail (e.g., data contain potentially sensitive information, data are owned by a third-party organization, etc.) and who has imposed them (e.g., an ethics committee). Please also provide contact information for a data access committee, ethics committee, or other institutional body to which data requests may be sent.***

***b) If there are no restrictions, please upload the minimal anonymized data set necessary to replicate your study findings as either Supporting Information files or to a stable, public repository and provide us with the relevant URLs, DOIs, or accession numbers. For a list of acceptable repositories, please see http://journals.plos.org/plosone/s/data-availability#loc-recommended-repositories.***

The above points have been included in the cover letter where there are no legal restrictions to share the data.

***4. Comment: Please ensure that you refer to Figure 1 in your text as, if accepted, production will need this reference to link the reader to the figure.***

 Figure 1 is now referred to in the text. Kindly see lines 244-245 (track changes showing). All figures now are referred to in the text.

**5. Comment: Please include a separate caption for each figure in your manuscript.**

A separate caption is now included for each figure.

**Reviewer #1:**

The authors would like to thank Reviewer 1 for the insightful comments. Indeed, the comments helped improved the paper a lot.

***1. Comment 1: English editing of the manuscript is required.***

The authors agree that English language is wordy in some sections, and therefore, english editing has now been done throughout the manuscript. For instance, kindly see:

- The Abstract
- Introduction
- Lines 365-369
- Lines 376-382
- Lines 391-394
- Lines 405-411
- Lines 424-428
- Conclusion and Implication part
- And other sections of the document.

***2. Comment: The abstract in the system and manuscript are not similar, please check carefully.***

They are updated now to be the same

***3. Comment: Line 243: UCINET is not explained, full form could be presented on its first appearance in the manuscript.***

Surprisingly, there is no full form for “UCINET”. UCINET is known as a windows software package for social network analysis (see <https://sites.google.com/site/ucinetsoftware/home>). The authors now included an explanation for UCINET in the manuscript (see lines 264-266)

***4. Comment: All abbreviation / acronyms could be thoroughly checked in the revised version.***

The main abbreviations are STM and SNA:

The abbreviation used for “Stakeholder theory and management” is “STM”. Now it has been used to replace the full word in line 33.

The abbreviation used for “social network analysis” is “SNA”. Now it has been used to replace the full word in lines:

114, 122, 179, 263, 264,802

There are no other abbreviations in the text.

***5. Comment: ED, the ego-density could be mentioned in preceding paragraphs.***

Kindly note that “ego-density” is mentioned in the abstract (line 44), introduction (124), literature (line 179) and in the remaining sections of the paper.

If it is requested that the abbreviation “ED” for “ego-density” to be used instead, the authors would like to kindly note that this can be done, however, they prefer that the full term “ego-density” to be used in conjunction with the other social network measures that have no abbreviations, such as betweenness, closesess, efficiency, etc. Otherwise, the authors have to find an abbreviation to all of these, which perhaps, might make it harder for the reader to memorize all of these terms while reading the paper.

***6. Comment: Discussion with reference to other studies and findings could have been added to strengthen the inferences derived from the analysis*.**

The results of this study are divided into 3 main section – authors, institutions and countries. Therefore, we have now referenced studies that support/contradict our findings for each of the three sections.

For authors, see: (the numbers of references changes, see again)

- Reference number 115, line 394
- Reference number 18, line 409
- Reference number 116, line 427

For institutions, see:

- Reference 16 line 470
- Reference 20, line 492
- Reference 20, line 503

For countries:

See references 20,142,144 in line 509

***7. Comment: Fig 2 the sharp dip in 2020 indicates that authors did not consider the whole year, I would suggest either remove 2020 or add the latest papers as well. Though, there is a possibility of decrease in number of publication due to the pandemic.***

The authors would like to thank the reviewers for this unintentionally overlooked mistake. The authors would like to note that they submitted this paper on November 24, and haven’t taken into consideration the published papers in December. After a review of all the papers published in 2020 (see picture below), the number increased from 256 to 356. So the final number is 356. The authors believe that the drop in number from 1163 to 356 is due to the Covid – 19 pandemic, which they personally experienced and has also impacted their publication performance overall as well as that of others.

**Reviewer #2:**

The authors would like to thank Reviewer 2 for the insightful comments. Indeed, the comments helped improved the paper a lot.

***1. Comment: Abstract needs revision in terms of methodology and possible practical policy implications at regional and global levels and in terms of language.***

***These changes have now been made:***

For revision in terms of methodology, see lines 28-35 (track changes showing)

For revision in terms of practical policy, see lines 45-53

Language has been improved by reducing the number of words and using more straightforward terminologies across the entire abstract, and also across the entire manuscript.

***2. Comment 3. Arrange keywords in alphabetical order***

Done

***3. Comment: Brief history section should be literature review section. And more sophisticatedly arranged.***

- “Brief history” is now replaced by” Literature Review”.
- The subsection “Origins of STM” has been included below “Literature Review”
- New subsection “Expansion of STM” has been included with new ideas about stakeholder analysis expansion (lines 151 and 155).
- Subsection “social network Theories” has been included within the “literature review” section

***4. Comment: How to validate data or verify because no specific source except WoS provided?***

The authors would like to kindly note that data, in such form of research, cannot be validated using traditional research validation techniques. The data is obtained from Web of Science (WoS) which is a trusted public source and is widely regarded by scholars as fairly comprehensive and robust. The same approach used in this study can be used to extract data from other scholarly databases like Scopus, Dimensions etc. However, there is overlaps among such data sources and WoS is the pioneer and most trusted source for meta-data analysis of academic articles although it is coverage may be less than other, focusing mainly on prominent journals (publishers).

***5. Comment: Why social network analysis is used? How about other methods?***

The reason why social network analysis research has been used in this study is because relationships are of paramount importance in explaining behavior, and in particular, how research performance is affected by the structure of authors’ relationships. We rely on a “networks perspective” that uses individual relations to explain individual outcomes. Also, social network analysis has been the main tool for exploring scientific collaboration networks in many studies (kindly see reference numbers 1,2,4,6,12,18,20 etc)

Regarding other methods, there is a wealth of social network models that can be utilized, but the choice boils down to which method addresses the papers’ aims:

*(i) explore the evolution of research collaboration networks, (ii) identify the key actors (authors, institutions and countries) that are leading collaborative works in each sub-period, and (iii) understand the longitudinal effect of co-authorship networks on research performance*

These aims require social network analysis to be used in order to **quantify** the structure of relationships

There are many social network methods in literature, and therefore, the authors have identified a number of well-known models that have been used but cannot be operationalised for this study. Among the popular network models is Exponential Random Graph Models (ERGM) that explores if a network deviates significantly from chance. ERGM requires that the social network is treated as the dependent variable, where in our study, the network constructs were treated as the independent variables and research performance as the dependent variables. Nevertheless, ERGMs requires incorporate nodal attributes in model estimation, where in our study, the characteristics of scientists were excluded. With respect to models that treat time more explicitly in the sense that they model what happens at the micro (individual) level at each point in time is network exposure which explores the social influence in diffusion, a topic that also falls out of scope. Kindly note that there are also other models that cannot be operationalized in this study, but the authors are afraid that discussing them would diverge the study away from its objective.

***5. Comment: Table captions are always provided at top of the table. Correct all.***

Done now for all 6 Tables.

***6. Comment: Conclusions and policy implications is way long section. Reduce it to 1.5 page only please. Only provide the most significant things.***

The authors reduced the number pages from 6 to less than 2.5 pages. The authors would like to kindly ask, if possible, to have 2.5 pages instead of 1.5, because we have too many results that need to be summarized (author, institution, countries, association testing) and there is also the theoretical and practical implications that need to be included as well.. Because it is a long manuscript so to speak, and has different research objectives, its better to have a conclusion that discusses briefly each objective:

- the evolution of collaboration networks for authors, instituions and countries.
- associated testing between network variables and research performance.
- we also include a paragraph about key words which is important, and associated figures.
- practical contribution.

***7. Comment: Fig. 4, network B, contains very minute words inside. Please use better font size.***

Larger font size is used now. Old figure deleted.

***8. Comment: Try not to use references older than 2012. And also add some citations from this journal PLOS One, to link your paper with this.***

The authors would like to kindly note that since we are covering an era between 1989 and 1999, and between 2000 and 2010, we had to include studies published in that era. However, these have been minimized now, and other new references, related to PLOS one and other journals, have been included/

References added (some are PLOS ONE)

- Jappe A. Professional standards in bibliometric research evaluation? A meta-evaluation of European assessment practice 2005–2019**. PloS one**. 2020;15(4):e0231735.
- Walker, J., Chaar, B. B., Vera, N., Pillai, A. S., Lim, J. S., Bero, L., & Moles, R. J. (2017). Medicine shortages in Fiji: A qualitative exploration of stakeholders’ views. ***PLoS One***, *12*(6), e0178429.
- Naseem MA, Lin J, Rehman RU, Ahmad MI, Ali R. Moderating role of financial ratios in corporate social responsibility disclosure and firm value. **PloS one.** 2019;14(4):e0215430
- Jensen MC. Value maximisation, stakeholder theory and the corporate objective function. Unfolding Stakeholder Thinking: Routledge; 2017. p. 65-84. Sartas, M., Van Asten, P., Schut, M., McCampbell, M., Awori, M., Muchunguzi, P., ... & Leeuwis, C. (2019). Factors influencing participation dynamics in research for development interventions with multi-stakeholder platforms: A metric approach to studying stakeholder participation. ***PloS one***, *14*(11), e0223044.
- LeClair, A. M., Kotzias, V., Garlick, J., Cole, A. M., Kwon, S. C., Lightfoot, A., & Concannon, T. W. (2020). Facilitating stakeholder engagement in early stage translational research. ***PloS one***, *15*(7), e0235400.
- Zweigenthal, V. E., Pick, W. M., & London, L. (2019). Stakeholders’ perspectives on Public Health Medicine in South Africa**. *PloS one***, *14*(8), e0221447
- Political stakeholder theory: The state, legitimacy, and the ethics of microfinance in emerging economies. Business Ethics Quarterly. 2017;27(1):71-98.
- Freeman RE, Dmytriyev S. Corporate social responsibility and stakeholder theory: Learning from each other. Symphonya Emerging Issues in Management. 2017(1):7-15.

**Old References removed:**

- Katz JS, Martin BR. What is research collaboration? Research policy. **1997**;26(1):1-18.
- De Haan J. Authorship patterns in Dutch sociology. Scientometrics. **1997**;39(2):197-208.
- Smith HJ. The shareholders vs. stakeholders debate. MIT Sloan Management Review. **2003**;44(4):85-90.
- Wallace JS. Value maximization and stakeholder theory: compatible or not? Journal of Applied Corporate Finance. **2003**;15(3):120-7.
- Zingales L. In search of new foundations. The journal of Finance. **2000**;55(4):1623-53.
- Orts EW, Strudler A. The ethical and environmental limits of stakeholder theory. Business Ethics Quarterly. **2002**:215-33.
- Laband DN, Tollison RD. Intellectual collaboration. Journal of Political economy. **2000**;108(3):632-62.
- Vučković-Dekić L. Authorship-coauthorship. Archive of Oncology. **2003**;11(3):211-2.
- Berman SL, Wicks AC, Kotha S, Jones TM. **1999**. Does stakeholder orientation matter? The relationship between stakeholder management models and firm financial performance. Academy of Management journal
- Jones TM. Instrumental stakeholder theory: A synthesis of ethics and economics. Academy of management review. **1995**;20(2):404-37.
- Jones TM, Wicks AC. Convergent stakeholder theory. Academy of management review. **1999**;24(2):206-21.
- Borgatti SP. Centrality and AIDS. Connections. **1995**;18(1):112-4
- Boiko PE, Morrill RL, Flynn J, Faustman EM, Belle Gv, Omenn GS. Who holds the stakes? A case study of stakeholder identification at two nuclear weapons production sites. Risk Analysis. **1996**;16(2):237-49
- Chubin D, Friedman R, Kemp K, Fainberg A, Linsenmeyer J. Policy analysis at the US Office of Technology Assessment. International Journal of Technology Management. **1996**;11(5-6):589-603.
- Harrison JS, Freeman RE. Stakeholders, social responsibility, and performance: Empirical evidence and theoretical perspectives. Academy of management Journal. **1999**;42(5):479-85.

**9. Comment: Lastly, English language editing required. Please avoid use of “we”, “our”, “us” in n**

English language has been edited throughout the paper and all “We”s have been removed (see lines: 25, 30, 111, 119, 126, 223, 282, 312, etc.

To see the English editing in some sections, kindly see below:

- Abstract
- Introduction
- Lines 365-369
- Lines 376-382
- Lines 391-394
- Lines 405-411
- Lines 424-428
- Conclusion and Implication part
- And other sections of the document.
